# The Role of Pentacam Random Forest Index in Detecting Subclinical Keratoconus in a Chinese Cohort

**DOI:** 10.3390/diagnostics14202304

**Published:** 2024-10-17

**Authors:** Yan Liu, Yu Zhang, Yuexin Wang, Ruilan Dong, Yueguo Chen

**Affiliations:** 1 Department of Ophthalmology, Peking University Third Hospital, 49 North Garden Road, Haidian District, Beijing 100191, China; doctorliuyan@bjmu.edu.cn (Y.L.); zhangyured@sohu.com (Y.Z.); wyx19920330@126.com (Y.W.); 18810589919@163.com (R.D.); 2 Beijing Key Laboratory of Restoration of Damaged Ocular Nerve, Peking University Third Hospital, Beijing 100191, China

**Keywords:** keratoconus, Pentacam random forest index (PRFI), tomography, Chinese refractive surgery candidates

## Abstract

**Purpose:** This study aimed to evaluate the diagnostic accuracy of a novel shape index, the Pentacam Random Forest Index (PRFI), in detecting keratoconus (KC), specifically subclinical keratoconus, in Chinese refractive surgery candidates. **Methods:** This prospective cohort study included 856 participants who were divided into four groups based on their tomographic outcomes: the KC group (*n* = 137), the very asymmetric ectasia (VAE) group (*n* = 73), the normal cornea group (*n* = 363) and the tomographically suspected KC (TSK) group (*n* = 283). The diagnostic performance of PRFI and other widely used indices, including the shape index BAD-D and the combined index TBI, was assessed using receiver operating characteristic (ROC) curve analysis and compared using DeLong’s test. The area under the curve (AUC), best cutoff values, and Youden index for each parameter are reported. Additionally, the false-positive rates of BAD-D and PRFI were calculated and compared in “normal corneas”. **Results:** All shape and biomechanical parameters collected in this study were found to be significantly different among the four groups (KC, VAE, TSK, and normal groups; *p* = 0.000). The AUC of PRFI was the highest in detecting any form of KC (including clinical KC eyes and VAE-NT eyes) in Chinese refractive surgery candidates, outperforming the widely used shape index BAD-D (0.919 vs. 0.890, *p* < 0.001). There was no significant difference in performance between the PRFI and the combined TBI index (0.919 vs. 0.916, *p* > 0.05). For detecting subclinical KC eyes (i.e., VAE-NT), the AUC of PRFI was 0.774, which was statistically comparable to TBI (0.774 vs. 0.776, *p* > 0.05), while outperforming BAD-D (0.774 vs. 0.684, *p* < 0.001). The best cutoff values of PRFI for detecting any KC and VAE-NT eyes were determined to be 0.37 and 0.27, respectively. Additionally, PRFI demonstrated a lower false-positive rate than BAD-D (13.8% vs. 43.8%, *p* < 0.001). Notably, the relatively high false-positive rate of BAD-D observed in this study might be attributed to the smaller horizontal corneal diameter in tomographically suspected eyes. **Conclusions:** The PRFI proved to be a superior shape index compared to BAD-D in detecting any form of keratoconus, including subclinical cases, in Chinese refractive surgery candidates. This finding may be attributed to the relatively small corneas commonly observed in Asians.

## 1. Introduction

In recent years, there have been significant advancements in the development of instruments for clinical detection and diagnosis of keratoconus (KC) [1,2,3]. The widespread use of tomography and corneal biomechanical analyzers has greatly improved the diagnostic rate of KC, leading to better prognosis and increased safety in refractive surgery [4,5]. Among the various tomographic devices available, the Pentacam^®^ HR (OCULUS Optikgeräte GmbH; Wetzlar, Germany) has proven to be a valuable tool for detecting early KC and monitoring its progression [1,2,6,7]. The Belin–Ambrósio deviation index (BAD-D) is a computed index developed to assess the predisposition to keratoconus using Pentacam^®^ HR parameters. It derives from a combination of ‘D’ values obtained through logistic regression analysis aimed at optimizing ectasia detection [7]. Many studies have reported that the BAD-D is a valuable parameter with relatively high sensitivity and specificity for detecting corneal ectasia [8,9,10]. However, in the Chinese population, we observed that BAD-D can sometimes be overly sensitive, which might easily suggest suspicious morphological abnormalities, especially in patients with smaller corneas. This has resulted in a relatively high false-positive rate [11,12]. Consequently, its clinical application in screening potential candidates for refractive surgery has been limited in this population.

Despite significant progress in corneal shape and biomechanical measurements, the accurate diagnosis of KC, especially in its earliest stages, remains a challenge [13,14,15]. In clinical practice, there are instances where the BAD-D index may fall within the ‘yellow’ range, indicating a potential risk for keratoconus; however, the surgeon, based on their experience and clinical judgment, decides whether the patient is safe to undergo refractive surgery. The decision-making process involves considering various factors such as the patient’s demographic information, corneal thickness, biomechanical stability, tomographic patterns, and other clinical indicators, in addition to the BAD-D index. In these cases, we found that most, if not all, of the patients who underwent corneal refractive surgery despite having a ‘yellow’ BAD-D range remained stable after two years of the corneal laser procedure. This suggests that in some instances, the BAD-D index itself may have a higher false-positive rate, leading to unnecessary concerns for patients and potentially excluding them from the opportunity to undergo corneal refractive surgeries.

While it is crucial to be cautious about false-negatives to avoid the development of secondary corneal ectasia and to ensure surgical safety, false-positives, where patients are misclassified as having potential keratoconus, can also have consequences. Patients classified as TSK based on tomographic findings may lose the opportunity for corneal refractive surgery, which could have otherwise been beneficial for vision correction. Additionally, these patients may undergo further examinations and invest in unnecessary expenses, causing an increased financial burden and inconvenience. The balance between avoiding false-negatives and minimizing false-positives in the detection of keratoconus and patient selection for refractive surgery presents a challenge. This underscores the importance of combining clinical judgment, individual patient characteristics, and additional diagnostic measures in making informed decisions. Ongoing research and improvements in technology and assessment methods aim to enhance the accuracy and reliability of keratoconus detection, reducing both false-negatives and false-positives, to ensure optimal surgical safety and outcomes for patients.

The Pentacam random forest index (PRFI), developed by Lopes et al. in 2018, has shown promising results in enhancing early ectasia diagnosis and reducing false-positives when screening refractive surgery candidates [16]. The PRFI algorithm uses a random forest artificial intelligence model generated using multiple Pentacam parameters. In a study conducted by Lopes et al., PRFI demonstrated an excellent diagnostic performance. When assessing all ectasia cases, an area under the curve (AUC) of 0.992 was achieved, with a sensitivity of 99.4% and specificity of 98.8%. Specifically, when it came to the detection of post-LASIK ectasia (PLE), PRFI also exhibited superior performance compared to the BAD-D index. It achieved an AUC of 0.966, with a sensitivity of 80% and a specificity of 96.8% [16]. Based on these results, the authors concluded that implementing PRFI could enhance the early ectasia diagnosis. Additionally, in our clinical practice with a majority of Chinese subjects, we found that PRFI is a very helpful parameter for reducing the false-positive rate of the BAD-D index when screening refractive surgery candidates. This implies that incorporating PRFI into the evaluation process may aid in selecting appropriate candidates for refractive surgery and minimizing unnecessary reexaminations.

As the number of corneal refractive surgeries has increased sharply in recent decades in China, detecting subclinical KC and predisposition to corneal ectasia preoperatively is of paramount importance to avoid surgical complications [17,18]. Because accurate diagnosis of subclinical KC remains a significant challenge for refractive surgeons, novel models and algorithms (including artificial intelligence and machine learning) should be developed and tested to improve diagnostic accuracy. This study aimed to determine the diagnostic ability of PRFI in distinguishing potential KC from normal eyes, especially in early forms of subclinical KC with no symptoms or signs.

## 2. Methods

### 2.1. Study Design

This study is a diagnostic test that includes participants from a prospective cohort.

### 2.2. Participants and Procedure

The study sample was sourced from the Peking University Third Hospital Ectasia Cornea Disease Cohort Project. This is a single-center prospective cohort study based on the outpatient population of Peking University Third Hospital in Beijing, China. The Third Hospital of Peking University is a renowned referral teaching institution. The Cohort Project was established in 2013 and focuses on patients who sought refractive surgery or corneal ectasia treatment at the Peking University Third Hospital Eye Center and consented to participate in the cohort study. 

All participants underwent a comprehensive ocular examination, which included assessments of uncorrected and corrected distance visual acuity, Jaeger near vision, slit-lamp examination, indirect ophthalmoscopy, corneal fluorescence staining, measurement of dark room pupil size, and manifest refraction with a phoropter. We also collected demographic information such as age, sex, history of refractive error, ocular surgery history, and family history for all participants. Auxiliary examinations were conducted as needed, including IOL Master (Carl Zeiss Meditec, Jena, Germany), Sirius (Costruzione Strumenti Oftalmici, Florence, Italy), Pentacam^®^ HR (Oculus, Wetzlar, Germany), Allegro Topolyzer (Alcon Laboratories, Inc., Fort Worth, TX, USA), and Anterior Segment OCT (Zeiss, Germany). All examination results were analyzed by Professor Yueguo Chen, who specializes in corneal disease and refractive surgery, and appropriate therapeutic management decisions were made based on this analysis. All patients were monitored for a minimum of 2 years, and necessary examinations were performed during each visit. An assigned employee was responsible for collecting all data files at baseline and follow-up, and the data were entered into Microsoft Excel 2019 by two assistants. 

### 2.3. Ethical Approval

This study was approved by the Ethics Committee of the Peking University Third Hospital (ethical approval No. M2023257, date 23 April 2023). All study procedures were conducted in accordance with the Declaration of Helsinki and written informed consent was obtained from all participants (and their parents or legal guardians if the participants were the under 18s) prior to enrollment.

### 2.4. Inclusion Criteria

Four types of patients from the cohort were included in this study:

I. Frank keratoconus (KC) eyes: These were eyes with typical clinical manifestations of KC as diagnosed by the professor in both eyes. One eye from each participant was included in the study.
II. Very asymmetric ectatic eyes with normal topography (VAE-NT) [19]: This group comprised patients with highly asymmetric ectatic eyes. When one eye was diagnosed with clinical KC, the other eye with normal topography was categorized as VAE-NT. The detailed criteria for VAE-NT are as follows:
  (1) Corrected distance visual acuity (CDVA) ≥20/20 Snellen equivalent (≤0 Logarithm of the Minimum Angle of Resolution [LogMAR]).
  (2) Normal topography was determined using an Allegro Topolyzer (WaveLight Technology AG, Alcon Laboratories, Erlangen, Germany) with a KC grading of KC0.
  (3) Mean keratometry (K) value <47 D and an inferior-superior (I-S) value ≤1.4 D according to the Rabinowitz and McDonnell criteria [19].
  (4) Pachymetry at the thinnest location >470 μm.
  (5) No signs of KC were observed on slit-lamp examination, and no central/paracentral or inferior focal steepening (anterior and/or posterior) and/or corneal thinning was observed.
  (6) Confirmed diagnosis of KC in the fellow eye.

**Figure 1:**
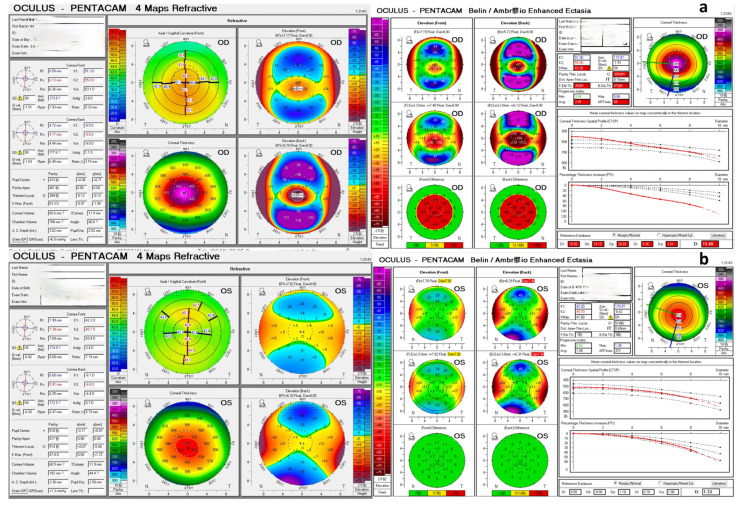
Typical tomography of both eyes of a patient with very asymmetric ectatic (VAE) eyes. (**a**) Demonstrates the tomography of the ectatic eye and (**b**) demonstrates the tomographically normal eye (VAE-NT).

A typical tomography of both eyes of a patient with VAE eyes was shown in Figure 1.

III. Normal eyes: This group comprised a consecutive series of patients who underwent corneal refractive surgery and were followed up for at least 2 years. These individuals showed no symptoms or signs of corneal ectasia and had completely normal findings on tomography.
IV. This group included consecutive cases with eyes marked as suspected BAD-D (marked with yellow) prior to refractive surgery but demonstrated safety and stability for at least 2 years postoperatively.

The exclusion criteria were previous ocular surgery or trauma history, significant corneal scarring, or associated ocular pathology. None of the participants wore soft contact lenses for 2 weeks or rigid gas permeable (RGP) lenses 3 weeks before the examinations; otherwise, they were excluded. Eyes with incomplete baseline information or those without follow-up records were also excluded.

### 2.5. Statistical Analysis

The statistical description included the calculation of the mean, standard deviation, and 95% confidence intervals. The normality of the data distribution was assessed using the Kolmogorov–Smirnov goodness-of-fit test. Data following a normal distribution were compared using one-way analysis of variance (ANOVA); otherwise, they were compared using the non-parametric Kruskal–Wallis test between groups. The Bonferroni test and post-hoc test for the Kruskal–Wallis analysis were used for pairwise comparisons. Receiver operating characteristic (ROC) curves were used to illustrate the sensitivity and specificity for different cutoff points of the corneal morphological and biomechanical parameters in KC, VAE-NT, normal eyes, and TSK eyes. Moreover, the best cut-off value, area under the ROC curve (AUC), and Youden index for PRFI, BAD-D, and TBI were determined. An AUC value of 1.0 indicates perfect discrimination, whereas values less than 0.5 show that the assessed parameter has no diagnostic ability. Pairwise comparisons of AUCs were performed using the DeLong test. The false-positive rate was calculated using the BAD-D cut-off value of 1.60 given by the device manufacturer. A cut-off value 0.27 of PRFI was adopted for detecting subclinical KC (VAE-NT eyes) in this study because no widely acknowledged cut-off value was available. The numerator was the number of cases whose BAD-D or PRFI exceeded the cut-off value; its denominator was 646 normal controls in our study. A Chi^2^ test was performed to compare the false-positive rates of BAD-D and PRFI. Statistical significance was defined as *p* < 0.05. All eligible data were analyzed using the IBM SPSS Version 23 statistical software (IBM SPSS Inc. Chicago, IL, USA) and the R statistical package (http://www.R-project.org (accessed on 20 July 2024); R Foundation for Statistical Computing, Vienna, Austria).

### 2.6. Incorporation of PRFI in Corneal Ectasia Screening

When screening corneal refractive surgery candidates, multiple factors should be taken into consideration, including but not limited to demographics and corneal properties. Other than widely used shape parameters such as BAD-D, PRFI was valuable in indicating the risk level for post-operative ectasia development. The PRFI values were categorized into low or high risk, based on the cutoff values established by Lopes et al. [16] or this study. These categories were used to stratify corneal refractive surgery candidates and to guide subsequent analyses. The PRFI scores were integrated with other clinical parameters to form a comprehensive assessment of each patient’s risk profile. This multifactorial approach allowed for a more nuanced understanding of the potential risks associated with refractive surgery.

## 3. Results

A total of 856 participants were enrolled in this study. Among them, 137 individuals had clinical bilateral keratoconus (KC), 73 had very asymmetric ectatic eyes (VAE), 283 had tomographic suspect keratoconus (TSK) eyes that underwent laser vision correction (LVC) surgery and remained stable for at least 2 years, and 363 had normal corneas with normal tomography results. The average ages of the participants in the four groups were as follows: clinical bilateral KC group, 24.61 ± 6.17 years (range 12–45); very asymmetric ectatic eyes group, 24.37 ± 6.13 years (range 13–37); TSK group, 27.83 ± 7.03 years (range 17–48); and normal corneas group, 27.45 ± 7.14 years (range 17–46), respectively (*p* < 0.001). The male/female ratios in the respective groups were 97/40, 50/23, 65/218, and 149/214. Table 1 displays the refractive information, main shape, and biomechanical parameters of the four groups along with their comparisons. All parameters demonstrated significant differences among the four groups (*p* < 0.001). The pairwise comparisons revealed statistically significant differences for all parameters, except for spherical equivalent (SE), maximum keratometry (Kmax), and inferior-superior value (I-S), between normal eyes and VAE-NT eyes (*p* = 0.08, *p* = 0.485, *p* = 0.198, respectively). Regarding corneal diameter (horizontal), the TSK eyes exhibited a significantly smaller size than the other groups (11.33 vs. 11.70, 11.79, and 11.81, *p* < 0.001). However, no significant difference in corneal diameter was found between KC and VAE-NT eyes (11.79 vs. 11.81, *p* = 0.712).

### 3.1. ROC Curves of PRFI and BAD-D to Distinguish Any KC from Control Eyes

**Figure 2:**
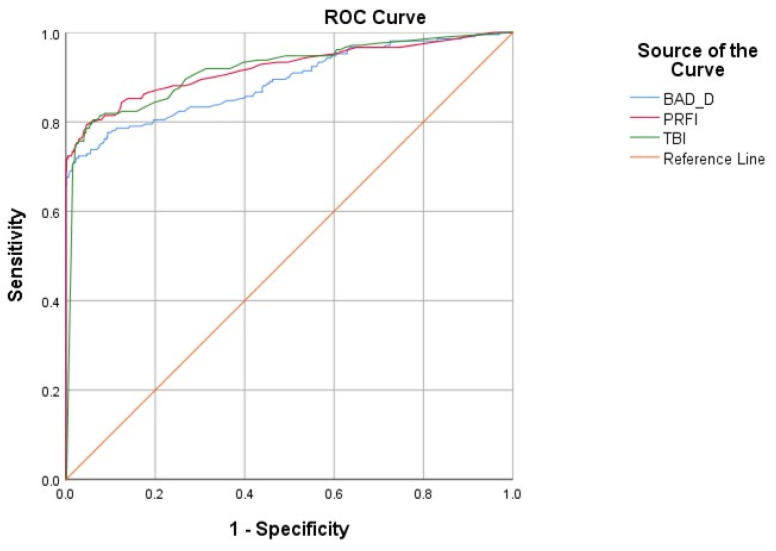
ROC curves of the Pentacam Random Forest Index (PRFI), Belin–Ambrosio-enhanced ectasia total deviation index (BAD-D) and Tomographic Biomechanical Index (TBI) in distinguishing any keratoconus (KC) eyes (*n* = 210) and control eyes (*n* = 646).

This study mainly focused on the two computed shape parameters using different algorithms (PRFI and BAD-D) to evaluate their receiver operating characteristic (ROC) curves and the best cut-off points for distinguishing KC eyes from control eyes. Considering the excellent performance of the combined index TBI in previous studies, TBI was also analyzed to compare the diagnostic accuracy. The ROC curves of PRFI, BAD-D, and TBI for distinguishing any KC eye (including both frank KC eyes and VAE-NT eyes) from control eyes are shown in Figure 2. The areas under the curve (AUCs), best cut-off points, Youden indices (Youden index = sensitivity + specificity − 1), sensitivity, and specificity are shown in Table 2. From these results, we found that the PRFI had a higher AUC than the BAD-D, reaching 0.919 (0.919 with a 95% CI of 0.893–0.946, vs. 0.890 with a 95% CI of 0.860–0.920, *p* < 0.001), which was comparable to that of TBI (0.916, 95% CI 0.890–0.942, *p* < 0.001). The comparison of AUCs (Delong test) showed that the AUC of PRFI was significantly higher than that of BAD-D (*p* < 0.001), with no significant difference from TBI (*p* > 0.05), indicating an outstanding diagnostic accuracy for the novel shape index PRFI. The detailed results of Delong’s test are shown in Table 3.

Furthermore, we noticed that when the PRFI cut-off value was established as 0.37, with a sensitivity of 79.5% and a specificity of 95.2% for detecting any form of KC eyes. The Yoden index of PRFI was higher than that of TBI and BAD-D, indicating the excellent performance of PRFI in detecting ectasic eyes in refractive surgery candidates. Moreover, when 1.60 was adopted as the BAD-D cutoff value given by the device manufacturer, the sensitivity and specificity were 86.7% and 57.1%, respectively.

### 3.2. ROC Curves of PRFI and BAD-D to Distinguish VAE-NT Eyes from Control Eyes

**Figure 3:**
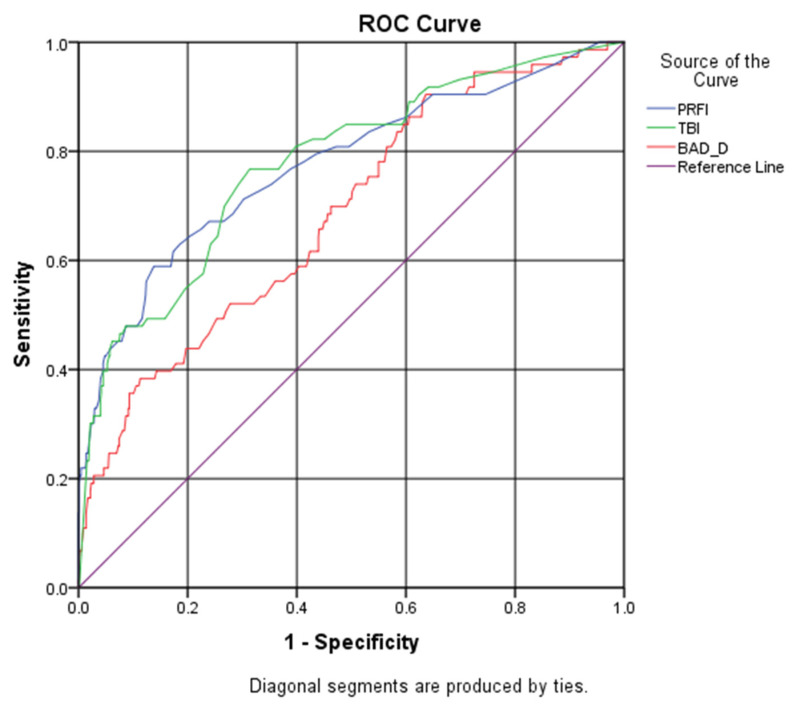
ROC curves of the Pentacam Random Forest Index (PRFI), Belin–Ambrosio enhanced ectasia total deviation index (BAD-D) and Tomographic Biomechanical Index (TBI) in distinguishing very asymmetric ectatic eyes with normal topography (VAE-NT) eyes (*n* = 73) and control eyes (*n* = 646).

As subclinical or early KC recognition and diagnosis is ambiguous and of great importance in clinical practice, we were more interested in identifying subclinical KC in refractive surgery candidates. Therefore, the ROC curves and best cut-off points were determined to differentiate VAE-NT (a classic form of subclinical KC) from control eyes [20,21], as shown in Figure 3 and Table 4. Similar to detecting any form of KC, PRFI had a higher AUC than BAD-D for VAE-NT eyes, reaching an AUC of 0.775 (0.775 with a 95% CI of 0.711 to 0.839 vs. 0.685 with a 95% CI of 0.620 to 0.749, *p* < 0.001). The AUC of the PRFI and TBI was also comparable (0.775 vs. 0.777, *p* = 0.8823), with Yoden indices of 0.451 and 0.453. This study showed that PRFI and TBI outperformed other parameters in detecting VAE-NT eyes, namely subclinical or early ectasic eyes, which indicated that the performance of this novel shape parameter PRFI has a diagnostic accuracy similar to that of the combined index, which considers both shape and biomechanical properties. The comparisons of the AUC of PRFI, BAD-D, and TBI in Delong’s tests are shown in Table 5.

Similarly, when the PRFI cut-off value was established as 0.27, the sensitivity for detecting VAE-NT eyes was 58.9% and the specificity was 86.2%, with a similar Yoden Index compared to TBI (0.451 vs. 0.453).

### 3.3. False-Positive Rate of BAD-D and PRFI

In the normal eye group, all patients underwent corneal laser surgery and remained stable for at least a 2-year follow-up period. We assume their corneas were actually “normal”, despite their BAD-D may be “yellow” or “red”. Specifically, when using the cut-off value of 1.60 provided by the machine manufacturer, 43.8% (283 out of 646) of refractive surgery candidates with normal corneas were evaluated as unsuitable for surgery due to elevated BAD-D values. In this study, the horizontal corneal diameter measured by Pentacam^®^ HR in TSK eyes was significantly smaller than that in other groups (11.33 vs. 11.70, 11.79 and 11.81, *p* < 0.001), which, to some extent, supported the perspective that the false-positive rate of BAD-D might be relatively high in Asians with smaller corneal sizes.

**Figure 4:**
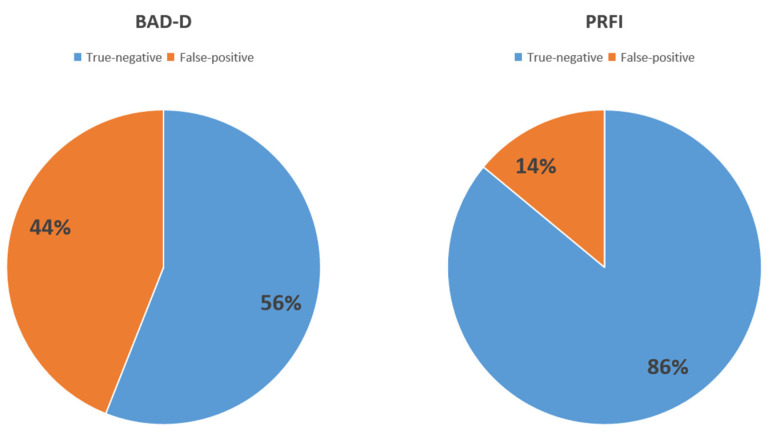
Distribution (false-positive rate) of the Belin/Ambrosio enhanced ectasia total deviation index (BAD-D) and Pentacam Random Forest Index (PRFI) in normal cornea group.

In contrast, PRFI showed promise in diagnosing corneal ectasia in individuals with smaller corneal diameters. In this study, a cut-off value of 0.27 was adopted for detecting subclinical ectasia. Using this threshold, only 13.78% (89 of 646) of surgical candidates were considered abnormal and potentially susceptible to corneal ectasia. The difference in false-positive rates between BAD-D and PRFI was found to be statistically significant (*p* < 0.001) using a chi-square test, as shown in Figure 4. These results suggest that PRFI may be a valuable shape index, particularly when dealing with individuals with smaller corneas, as it could potentially reduce the relatively high false-positive rate associated with BAD-D in this population. Overall, these findings highlight the potential benefit of incorporating PRFI as a diagnostic tool in identifying corneal ectasia, especially in individuals with smaller corneal sizes, and its potential to mitigate the false-positive rate observed with BAD-D in this population.

## 4. Discussion

It is of paramount importance to recognize any signs or predispositions to corneal ectasia prior to corneal laser surgery, as most iatrogenic ectasia results from preexisting unnoticeable ectasic conditions [22,23]. Due to the development and progress of tomography and in vivo biomechanical analysis, clinical KC is easy to diagnose and seldom missed. However, accurately differentiating subclinical KC, marked by ambiguous abnormalities in corneal shape and biomechanical properties, remains challenging [13,14,15]. Missed detection of potential corneal ectasia (false-negatives) might result in iatrogenic ectasia, causing unwanted vision loss in refractive surgery candidates, necessitating utmost vigilance. On the other hand, mistaking normal corneal structures for pathological ones in tomography (false-positives) could prevent surgery candidates from proceeding with operations and incur unnecessary expenses in their reexaminations. Therefore, an ideal screening strategy demands both high sensitivity and specificity, and novel techniques and algorithms, including artificial intelligence (AI) and machine learning technology, are now being applied in this field.

One issue is the inconsistency in findings due to the varied definitions of early or subclinical KC forms, such as suspected keratoconus (SCK), subclinical KC, or forme fruste KC (FFKC). In this study, VAE-NT eyes were included as a special type of subclinical KC group. This is because clinical KC had occurred in the fellow eyes, and according to the 2015 keratoconus global consensus, “true unilateral keratoconus does not exist” [24]. The VAE-NT eyes could be seen as an early form of KC without abnormal topography. However, subtle morphological alternations have already taken place quietly and might be detected using tomography and biomechanical assessment, especially when various advanced algorithms and techniques are introduced. As a result, we used VAE-NT eyes as representative of subclinical KC eyes, as in many previous studies [20,21,25]. More importantly, the diagnostic accuracy of the novel indices was tested to distinguish VAE-NT eyes from healthy eyes.

A previous study reported a relatively high false-positive rate in astigmatic eyes (astigmatism > 1.5 D) using Scheimpflug technology, and the false-positive rate varied depending on the cut-off values [13]. For BAD-D, false-positive rates could reach 100% at lower cut-off values; even at higher cut-off values, the false-positive rate for detecting potential ectasic corneal conditions could still remain at 57% [13], which might be nugatory or misleading for screening KC in refractive surgery candidates. In previous studies, reported factors related to “false-positives” of BAD-D were smaller corneal diameters in Chinese people [11,12], thinner corneas [11], obscure definitions of potential ectasic corneal conditions, and geographic and genetic characteristics of the population [13]. A previous study reported horizontal visible iris diameter (HVID) values measured by Sirius as 11.78 ± 0.37 mm and 12.04 ± 0.38 mm in tomography suspect KC group and control group, respectively (*p* < 0.001), indicating a higher false-positive rate in smaller corneas [11]. This phenomenon was also observed in the present study. The main purpose of this study was to improve the detection accuracy (decrease the false-positive rate of BAD-D) using a novel morphological computed index, and to determine a proper cut-off value.

In this study, we found that PRFI was superior to BAD-D for preoperative screening of KC in Chinese refractive surgery candidates, which was consistent with previous studies focused on North and South Americans and Europeans [16,26,27], as well as Asians [28,29]. The false-positive rate of PRFI was lower than that of BAD-D, indicating that PRFI could reduce the relatively high false-positive rate of BAD-D when recognizing KC, especially subclinical KC, in refractive surgery screening in the Chinese population. Moreover, according to Table 2, when the cutoff value of PRFI was set at 0.37, the sensitivity of detecting any form of KC was 79.5%, which was slightly lower than that of BAD-D with a 1.60 cut-off value (79.5% vs. 86.7%), but the specificity was significantly higher (95.2% vs. 57.1%). The same situation occurred when detecting VAE-NT eyes, with lower sensitivity (58.9% vs. 61.6%) and much higher specificity (86.2% vs. 57.1%). The Yoden index of PRFI was superior in both cases, indicating better diagnostic accuracy of PRFI than that of BAD-D. This result showed that PRFI had superior detection specificity, which was of great value in refractive surgery candidate screening.

It is worth noting that when distinguishing KC eyes from normal eyes, the AUC of the PRFI and TBI was comparable, with no significant difference (Delong’s test, *p* > 0.05). TBI—short for tomographic/biomechanical index—reflects a combination of corneal shape and biomechanical properties, which enables the robust integration of corneal morphology from the Pentacam^®^ HR and corneal biomechanics from the Corvis ST [20] and has shown excellent performance in previous studies [20,21,30,31]. This study found that PRFI was as useful as TBI in detecting any form of KC or subclinical KC (VAE-NT eyes). Although biomechanical information is essential for differentiating atypical or early forms of KC, the diagnostic accuracy of the novel shape index, the PRFI, was similar to the combined index, which is clinically very practical because biomechanical measurements are not always available in some circumstances. In summary, the situation faced in clinical applications is often very complex and requires problem-specific analysis. A proper combination of shape and biomechanical characteristics and comprehensive consideration are needed when a final diagnosis is made.

## 5. Limitations

Ideally, retrospective analysis of preoperative corneal conditions in iatrogenic ectasia cases would help identify predispositions to corneal ectasia and validate the most useful indices [16,18]. However, cases of post-surgical ectasia are rare in the clinic, and preoperative data are often incomplete and difficult to collect. Therefore, we used VAE-NT eyes as representative of subclinical or asymptomatic KC to test the screening accuracy of corneal ectasia. Future studies should include post-surgery ectasia cases to determine the sensitivity and specificity of PRFI and other indices. In this study, we included a true-negative group (normal eyes) with a follow-up period of at least two years. In other words, they remained stable for at least two years after corneal laser surgery, but there was still little possibility that they might develop iatrogenic ectasia in a longer follow-up period [32]. Therefore, we can only conclude with high probability that the corneas were normal, which might affect the study’s credibility.

Although PRFI had the highest AUC with a relatively satisfactory Yoden index among the indices tested, 41.1% of the VAE-NT eyes might have been missed when dealing with subclinical ectasic eyes (at a cut-off value of 0.27). If unrecognized in clinical settings, these patients could develop post-surgery ectasia. Other minor issues included non-comparable gender ratios and ages between groups, especially between the KC group and normal cornea subjects, due to the different characteristics of the two groups, which was difficult to avoid and was reported in the results. As a result, PRFI cannot be used clinically alone, and a comprehensive combination of other shape and biomechanical parameters, as well as age and gender, is needed to enhance detection sensitivity and specificity. Further investigation using novel models and algorithms (including artificial intelligence and machine learning) is warranted to improve screening efficacy.

## 6. Conclusions

PRFI proved superior in detecting any form of KC or subclinical KC over BAD-D in the Chinese population, potentially reducing the relatively high false-positive rate associated with BAD-D. Further studies are warranted, including post-surgery ectasia cases and longer follow-up periods, to improve the screening efficacy.

## Figures and Tables

**Table 1:** Shape and biomechanical parameters of KC, VAE-NT, TSK and normal eyes.

	KC Eyes		VAE-NT Eyes		TSK Eyes		Normal Eyes		*p*
	Mean	95% CI	Mean	95% CI	Mean	95% CI	Mean	95% CI	
SE (D)	−7.70 ± 4.01	−8.38 to −7.02	−4.76 ± 2.95	−5.46 to −4.06	−5.21 ± 1.69	−5.41 to −5.01	−5.55 ± 2.03	−5.76 to −5.34	<0.001
CCT (um)	470.38 ± 40.88	463.44 to 477.31	522.54 ± 34.38	514.46 to 530.62	533.56 ± 24.03	530.75 to 536.38	552.68 ± 28.98	549.68 to 555.69	<0.001
Pachy Min (um)	452.55 ± 43.82	445.15 to 459.96	517.15 ± 35.30	508.91 to 525.39	528.07 ± 23.94	525.27 to 530.87	548.14 ± 28.72	545.18 to 551.11	<0.001
Kmax (D)	57.77 ± 9.61	56.15 to 59.39	44.97 ± 1.66	44.58 to 45.36	45.72 ± 1.47	45.55 to 45.89	44.04 ± 1.49	43.89 to 44.20	<0.001
I-S value (D)	4.75 ± 3.30	4.19 to 5.31	0.59 ± 0.87	0.39 to 0.80	0.36 ± 0.61	0.29 to 0.43	0.13 ± 0.55	0.07 to 0.18	<0.001
Cornea Diameter (mm)	11.79 ± 0.40	11.71 to 11.86	11.81 ± 0.38	11.71 to 11.90	11.33 ± 0.36	11.28 to 11.37	11.70 ± 0.37	11.66 to 11.74	<0.001
BAD-D	9.30 ± 5.05	8.44 to 10.15	1.75 ± 0.67	1.60 to 1.91	1.91 ± 0.27	1.88 to1.94	0.75 ± 0.49	0.70 to 0.80	<0.001
PRFI	0.96 ± 0.09	0.94 to 0.98	0.36 ± 0.26	0.30 to 0.42	0.22 ± 0.13	0.21 to 0.24	0.06 ± 0.06	0.05 to 0.07	<0.001
CBI	0.98 ± 0.48	0.86 to 1.00	0.33 ± 0.31	0.26 to 0.40	0.20 ± 0.19	0.18 to 0.22	0.09 ± 0.11	0.08 to 0.10	<0.001
TBI	1.00 ± 0.04	0.99 to 1.00	0.54 ± 0.36	0.45 to 0.62	0.34 ± 0.22	0.32 to 0.37	0.10 ± 0.16	0.08 to 0.11	<0.001

KC, keratoconus; VAE-NT, fellow normal topography of very asymmetric ectasic cases; TSK, tomographic suspect keratoconus. 95% CI means 95% confidence interval; SE, spherical equivalent; CCT, central corneal thickness; Pachy Min, minimum corneal thickness; Kmax, maximum corneal front K value; BAD_D, Belin–Ambrósio deviation index; PRFI, Pentacam random forest index; CBI, Corvis biomechanical index; TBI, tomographic and biomechanical index. *p* was calculated to determine the differences between the three groups.

**Table 2:** AUC and best cut-off values for Distinguishing any KC Eyes (*n* = 210) from Normal Eyes. (*n* = 646).

	AUC	Cut-Off Value	Yoden Index	Sensitivity (%)	Specificity (%)
PRFI	0.919	0.37	0.747	79.5	95.2
BAD_D	0.890	2.40	0.696	72.4	97.2
TBI	0.916	0.55	0.743	80.5	93.8

**Table 3:** The pairwise comparisons of AUC of ROC curves when detecting all KC eyes (Delong test).

Item 1	Item 2	AUC Difference	95% CI	*z* Value	*p* Value
BAD_D	PRFI	0.029	0.014–0.045	3.68	0.0002 *
BAD_D	TBI	0.026	0.007–0.045	2.73	0.0064 *
PRFI	TBI	0.003	−0.009–0.015	0.55	0.5844

* represents a statistically significant difference in AUC.

**Table 4:** AUC and best cut-off values for Distinguishing VAE-NT Eyes (*n* = 73) from Normal Eyes (*n* = 646).

	AUC	Cut-Off Value	Yoden Index	Sensitivity (%)	Specificity (%)
PRFI	0.775	0.27	0.451	58.9%	86.2%
BAD_D	0.685	1.99	0.271	38.4%	88.7%
TBI	0.777	0.27	0.453	76.7%	68.6%

**Table 5:** The pairwise comparisons of AUC of ROC curves when detecting VAE-NT eyes.

Item 1	Item 2	AUC Difference	95% CI	*z* Value	*p* Value
BAD_D	PRFI	0.090	0.050~0.130	4.42	0.0000 *
BAD_D	TBI	0.092	0.044~0.141	3.73	0.0002 *
PRFI	TBI	0.002	−0.028~0.033	0.15	0.8823

* represents a statistically significant difference in AUC.

## Data Availability

The datasets generated during the current study are available from the corresponding author on reasonable request.
